# Is the Panel Doctor Doing His Job?

**Published:** 1922-04

**Authors:** R. W. Harris

**Affiliations:** (formerly Assistant Secretary, responsible for the Administration of National Health Insurance Medical Benefits, Ministry of Health)


### IS THE PANEL DOCTOR DOING HIS JOB?

**By R. W. HARRIS (formerly Assistant Secretary, responsible for the Administration of National Health Insurance Medical Benefits, Ministry of Health).**

"PANEL Doctor Drunk while Driving a Motor Car," was a headline in a leading evening newspaper some little time ago; and on another occasion: "Panel Doctor Charged with Grave Offence." I ask, in all seriousness, why "Panel" Doctor? It is conceivable that the writers felt that what would be a scandal in the case of any medical man becomes a grave scandal when that medical man is part of a Public Service. That, of course, is true, but I question very much whether it was in the mind of the writers of the headlines.

There is, unquestionably, a good deal of rather loose criticism of the panel doctor. He is spoken of, very often by people of some standing, as though he were a sort of inferior brand of medical practitioner. Now, I hold no brief for the panel doctor; he has very able representatives who are quite able to deal faithfully with his critics. But it is very desirable that all criticism should be based on an accurate knowledge of the facts, and, secondly, that a due sense of proportion should be observed. As to the facts, the truth is that the majority of medical men in general practice are, in fact, engaged in National Health Insurance practice, in greater or less degree. The patients comprise substantially the whole working population of the country. There are, of course, considerable exceptions and, in particular, all non-manual workers with an income of more than £250 a year are outside the scope of the Medical Benefit provisions of the National Health Insurance Acts. But, substantially, the working population of Great Britain receive treatment from their general practitioner under this Scheme, and they number roughly, fifteen millions.

Criticism in general terms, therefore, of the panel doctor would really amount to an indictment of the medical profession; and if "panel" treatment is an inferior form of general practitioner treatment, it is surprising that the whole working population make so comparatively few formal complaints. It would not, I think, be seriously suggested that the doctors who are in attendance on the insured population and who, broadly speaking, are attending their wives and families as private patients, are giving one sort of treatment to the insured person and another to his dependants.

What is, in fact, the standard of treatment which is expected of the panel doctor? When the Government and the doctors went to arbitration in the early part of 1920 on the question of remuneration, it was essential that there should be a clear understanding on both sides, and in the minds of the arbitrators, as to the standard of treatment to which the doctor was supposed to conform. From the Statement of Case made on behalf of the Government in the Arbitration proceedings, I quote the following:—

> "The maintenance in insurance practice of the best attainable standard of general practitioner efficiency has been the aim of the Government since the inception of the Insurance Medical Service. In a letter to the British Medical Association of November 6, 1912 (Cd. 6520, page 17) the then Chancellor of the Exchequer, Mr. Lloyd George, stated:—
>
> "'The standard of service which it is hoped that the doctors attending insured persons will reach and maintain is that which the best opinion in the profession itself would expect from a general practitioner in his ordinary work.'"

The doctors' representatives in the Arbitration proceedings urged for their part that "the remuneration of insurance practice must not be based on the lowest level of private practice, but on a relatively high level." It was, therefore, common round that the insurance practitioner must observer at least the standard to which he would conform if he were, in fact, treating his insurance patients as paying patients—as indeed they really are, the only difference being that their contract with the doctor takes the form of a capitation payment, whether the insured person is well or ill, instead of a special payment for services rendered at the time of illness.

I propose in a later article to examine more closely the bearing of this matter of remuneration on the quality of the service which may be expected from a general practitioner; and also how far it is necessary to an effective service that some limit should be placed on the number of insured persons that any one doctor should be allowed to have on his list. In the meantime it is perhaps sufficient to say that I do not think that the insurance medical service is in any way prejudiced either by the rate of remuneration or by the number of patients that any one doctor is allowed to accept.

The service is prejudiced and, unfortunately, badly prejudiced by individual cases of laxity, of irregularity in giving certificates, of actual neglect of patients, and, sometimes, by the improper acceptance of fees from insured persons, this last being a matter about which it would be difficult to write with moderation.

In a service which every registered general practitioner as such has a right to enter, and in which, in fact, some 12,000 general practitioners are engaged, there are bound to be a certain number of black sheep in the fold. It is far easier to make general statements about the service as a whole, whether on a public platform or in the columns of the Press, than to take bad cases of default and carry them before the appropriate tribunal. General statements of a sweeping character are not merely unfair to the great body of general practitioners who are engaged from day to day in, as I believe, putting their best into their insurance work as into the rest of their practice, but they tend to surround the whole service with an unfavourable atmosphere which must inevitably react to some extent on the treatment which the insured person receives. Is, then, the panel doctor to be exempt from criticism? Far from it. It is essential in the interests of the gradual improvement of the service that the bad doctor should be eliminated and that the indifferent doctor or the careless doctor should have brought home to him the necessity for the faithful observance of his contract. To this end there exists the necessary disciplinary machinery, which is not the mere product of the official mind but has been created and amended in close consultation with the representatives of the doctors themselves.

The question asked in colloquial phrase at the head of this article is not one which can be fully examined within the limits of a single article. It is necessary to the further examination of the question that the nature of the insurance doctor's contract should be clearly understood, and also that the question should be kept quite distinct from that of whether the insurance medical service is a satisfactory one—a much larger question, which involves wider considerations. There are deficiencies of the insurance medical service which are largely a matter of lack of funds. The panel doctor is working, in effect, with one hand tied behind his back, and until the service as a whole can be developed in various ways, to which I may perhaps later be allowed to make reference, it is clear that the service must always expect to receive a good deal of criticism, even though every doctor on the panel were giving to the insurance medical service the best of which he is capable.

### LORD KNUTSFORD'S WORK AT THE LONDON HOSPITAL.

On March 6 an address was presented to Lord Knutsford by the President, Vice-Presidents, House Committee, and hon. medical and surgical staff, to mark the completion of the twenty-fifth year of his chairmanship of the London Hospital and appreciation of his services to that institution. Mr. W. T. Paulin (treasurer) presided, and among those present were Lady Knutsford (to whom the nurses gave a bouquet), the Hon. Lucy Holland, Miss Paulin, Sir William Goschen, Sir Evan Spicer, Sir Hugh Rigby, Lord Dawson of Penn, Mr. Frank Curzon, Mr. Douro Hoare, Mr. John Henry Buxton, Sir William Lister, Mr. James Sherren, Colonel Openshaw, Dr. R. Hutchison, Mr. E. W. Morris (house governor), and Professor Bulloch.

The Chairman said that on December 7 last Lord Knutsford celebrated his silver wedding, and a few days later the House Committee re-elected him as their chairman for the twenty-sixth time. That great hospital, with its splendid equipment, its growth, the fine spirit that was in it, expressed better than any words could something of the work, the courage, the patience, the foresight, the imagination of the man whom so long ago they asked to be their leader. His chairmanship had been marked by the great rebuilding scheme, which had cost nearly £1,000,000, and gave them elbow room to do their work. Other additions, and newly-opened departments, arose as the direct result of the new movement for attacking disease; then communal medicine began to take definite shape, and now they had ante-natal clinics, children's clinics, and county council clinics in aural, ophthalmic, dental, and skin departments, and venereal clinics. The training of nurses was made more complete to keep them abreast with the times as medicine became more complex, and Tredegar House was built. In 1897 the King's Fund invited them to spend £100,000 of their capital on the improvement of the hospital. They had since spent ten times that sum, and the hospital stood to-day one of the finest centres in the world for the relief of the sick poor and for the training of nurses.

Lord Dawson, speaking on behalf of the medical and surgical staff, said that between the years 1896 and 1903 the hospital buildings were modernised and re-cast, operating theatres and equipment representing all that modern knowledge could devise were created, and thus rendered possible the high surgical skill for which the hospital was renowned. All these additions exemplified the alertness of the hospital under the chairman's inspiring leadership. This leadership found expression not alone in bricks and mortar, but in an infectious enthusiasm for medical progress and in determination that the London Hospital should be in the van of that progress. They rejoiced to think of that occasion not as a farewell, but a landmark of a great career. His enthusiasm, his vitality, his fun, and his sense of humour had played no small part in his success. Lord Knutsford had differed on varying occasions from most of them, but never once had he failed in generosity and consideration. A Prince of Beggars, he had the instincts of the sportsman and the genius of the friend.

The Chairman then asked Lord Knutsford to accept the address, together with a silver inkstand and ornaments. The address, which bore the signature of Queen Alexandra as President of the hospital, was as follows :—

"The President, Vice-Presidents, House Committee, and Honorary Medical and Surgical Staff of the London Hospital, on the completion of Lord Knutsford's twenty-fifth year of chairmanship, desire to place on record their appreciation of the great services rendered by him to the hospital during this quarter of a century. How great some of these services have been may be understood by walking round the hospital. It has been largely rebuilt and very much extended at a cost of nearly one million pounds—a wonderful achievement. But there are other services, services which cannot be estimated simply in material things—in increased wealth, in enlarged structure, or in improved equipment. We wish, therefore, to pay tribute to the fine spirit of the old hospital, and we lay this tribute before Lord Knutsford. When he came it was a shelter for the needy and outcast; at the end of 25 years it is a great fighting force against disease and all that makes men needy and outcast. But in gaining efficiency it has kept its charity, and we acknowledge that it is by the imagination of Lord Knutsford and his insistence that the hospital motto should be the rule of the hospital's life that this triumph has been achieved."

